# Nondirective meditation activates default mode network and areas associated with memory retrieval and emotional processing

**DOI:** 10.3389/fnhum.2014.00086

**Published:** 2014-02-26

**Authors:** Jian Xu, Alexandra Vik, Inge R. Groote, Jim Lagopoulos, Are Holen, Øyvind Ellingsen, Asta K. Håberg, Svend Davanger

**Affiliations:** ^1^Department of Medical Imaging, St. Olavs HospitalTrondheim, Norway; ^2^Department of Biological and Medical Psychology, University of BergenBergen, Norway; ^3^Department of Psychology, University of OsloOslo, Norway; ^4^Clinical Research Unit, Brain and Mind Research Institute, University of SydneySydney, NSW, Australia; ^5^Department of Neuroscience, Faculty of Medicine, Norwegian University of Science and TechnologyTrondheim, Norway; ^6^Centre for Pain and Complex Disorders, St. Olavs HospitalTrondheim, Norway; ^7^Department of Circulation and Medical Imaging, Faculty of Medicine, Norwegian University of Science and TechnologyTrondheim, Norway; ^8^Department of Cardiology, St. Olavs HospitalTrondheim, Norway; ^9^Department of Anatomy, Institute of Basic Medical Science, University of OsloOslo, Norway

**Keywords:** fMRI, meditation, attention, nondirective, brain, default mode network, mind wandering

## Abstract

Nondirective meditation techniques are practiced with a relaxed focus of attention that permits spontaneously occurring thoughts, images, sensations, memories, and emotions to emerge and pass freely, without any expectation that mind wandering should abate. These techniques are thought to facilitate mental processing of emotional experiences, thereby contributing to wellness and stress management. The present study assessed brain activity by functional magnetic resonance imaging (fMRI) in 14 experienced practitioners of Acem meditation in two experimental conditions. In the first, nondirective meditation was compared to rest. Significantly increased activity was detected in areas associated with attention, mind wandering, retrieval of episodic memories, and emotional processing. In the second condition, participants carried out concentrative practicing of the same meditation technique, actively trying to avoid mind wandering. The contrast nondirective meditation > concentrative practicing was characterized by higher activity in the right medial temporal lobe (parahippocampal gyrus and amygdala). In conclusion, the present results support the notion that nondirective meditation, which permits mind wandering, involves more extensive activation of brain areas associated with episodic memories and emotional processing, than during concentrative practicing or regular rest.

## Introduction

### Volitional and spontaneous activities in meditation

Many types of meditation used for stress management and health can be described as a cycle of volitional and spontaneous cognitive processes (Cardoso et al., 2004). Attention is intentionally focused on a suitable meditation object, such as mental repetition of a non-semantic meditation sound, sensations associated with breath or specific regions of the body, a physical or mental visual image, or by simply being aware of the shifting flow of inner experiences (Cardoso et al., 2004; Ospina et al., 2007). Focusing on the meditation object is typically interspersed with periods of mind wandering (Cardoso et al., 2004; Ospina et al., 2007; Hasenkamp et al., 2012), which has been defined as being absorbed in spontaneously occurring thoughts, images, sensations, memories, and emotions unrelated to current volitional activity, more or less without really being aware of it (Mason et al., 2007; Christoff et al., 2009). An example of this cognitive cycle is given in a detailed temporal study of meditation with focused attention on the breath (Hasenkamp et al., 2012). Functional magnetic resonance imaging (fMRI) was used to correlate brain activation with cognitive processes that describes the shifting between focusing on the meditation object and spontaneously occurring thought. Mind wandering was associated with activation of the default mode network as well as sensory and motor cortices and posterior insula. Becoming aware that the breath was completely out of the focus of attention was associated with activation of the salience network. Shifting back to the breath and sustaining the focus on it were associated with elements of the executive network (Hasenkamp et al., 2012).

### Different perceptions of mind wandering

The function of spontaneous mental processes in meditation is controversial. How they are dealt with, depends on the type of practice (Box 1–3). In most mindfulness practices and many other techniques associated with Buddhist traditions, mind wandering is considered a distraction and a gateway to rumination, anxiety and depression (Sood and Jones, 2013). An ultimate goal of these methods is therefore to reduce mind wandering and its purported negative consequences (Brewer et al., 2011; Sood and Jones, 2013; Taylor et al., 2013). In contrast, some practices consider the spontaneous flow of inner experiences as part of the meditation process. Accepting mind wandering while practicing is a core element in the Relaxation Response, Transcendental Meditation, Clinically Standardized Meditation, and Acem Meditation (Benson et al., 1975; Carrington et al., 1980; Carrington, 1998; Ospina et al., 2007; Davanger et al., 2010; Travis and Shear, 2010). As described below, these techniques may be classified as nondirective, indicating less control of the process while practicing (Box 3). It has been proposed that types of meditation that allow spontaneous thoughts, images, sensations, memories, and emotions to emerge and pass freely without actively controlling or pursuing them, over time may reduce stress by increasing awareness and acceptance of emotionally charged experiences (Ellingsen and Holen, 2008; Lutz et al., 2008a; Davidson, 2010). This notion concurs with recent articles suggesting that mind wandering and activation of the default mode network in general may serve introspective and adaptive functions beyond rumination and daydreaming (Ottaviani et al., 2013). Potentially useful functions would include mental simulations, using autobiographical memory retrieval to envision the future and conceiving the perspective of others (Buckner et al., 2008; Andrews-Hanna, 2012). An interesting question is therefore whether type of meditation and mode of practicing might affect the extent of mind wandering and the pattern of default mode activation during meditation.

Box 1 **Focused attention**

Focused attention practices usually entail paying attention to the physical sensation of the breath wherever it is felt most strongly in the body, without trying to change it in any way. Whenever attention has wandered to something else, the meditator gently but firmly brings it back to the physical sensation of the breath (Brewer et al., 2011). Important aims of the practice are to quickly detect mind wandering and maintain attention more stably on the breath, eventually needing less effort in the task, and over time reducing emotional reactivity (Lutz et al., 2008b). Focused attention practices typically involve a relatively narrow field of focus. As a result, the ability to identify stimuli outside that field of focus may be reduced (Lutz et al., 2008b).

Box 2 **Open monitoring**

Open monitoring practices (sometimes called choiceless awareness) are described as paying attention to whatever comes into ones awareness - whether it is a thought, emotion, or body sensation - just following it until something else emerges without trying to hold onto it or change it in any way (Brewer et al., 2011). Even though “effortful selection” or “grasping” of an object as primary focus is gradually replaced by “effortless sustaining of awareness without explicit selection,” the core activity of the practice is to sustain attention with the shifting flow of experiences, sometimes detecting emotional tone as a background feature (Lutz et al., 2008b).

Box 3 **Nondirective meditation**

In nondirective meditation practices, a relaxed focus of attention is established by effortless, mental repetition of a short sequence of syllables, which may either be a traditional mantra or a non-semantic meditation sound (Benson et al., 1975; Carrington et al., 1980; Ospina et al., 2007; Davanger et al., 2010; Travis and Shear, 2010). Whenever the meditator becomes aware that the focus of attention has shifted to mainly being occupied with spontaneously occurring thoughts, images, sensations, memories, or emotions, attention is gently and non-judgmentally redirected to repetition of the meditation sound. The aim of the practice is to increase the ability to accept and tolerate stressful and emotional experiences as a normal part of meditation as well as everyday life (Davanger et al., 2010). Attention is neither directed toward staying with the meditation object like in focused attention techniques nor directed toward observing the spontaneous flow of experiences like in open monitoring meditation (Lutz et al., 2008b). Consequently, such methods comprise a distinct style of practicing (Cahn and Polich, 2006; Ellingsen and Holen, 2008; Travis and Shear, 2010), that has previously been termed nondirective meditation, as the presence of spontaneously occurring thoughts, images, sensations, memories, and emotions is accepted without actively directing attention toward them or away from them (Ellingsen and Holen, 2008; Lagopoulos et al., 2009; Nesvold et al., 2011). Further details on Acem meditation and its background are provided in previous publications (Ellingsen and Holen, 2008; Davanger et al., 2010).

### Extent of mind wandering

It is often assumed that mind wandering is reduced during meditation, and more so in practitioners with many years of experience. The evidence comes from a relatively small number of studies in which the extent of mind wandering was assessed by questionnaire. Self-reported mind wandering during meditation was less abundant in participants with long-term experience in “concentration” (focused attention on breath), “loving-kindness meditation” (exercise oriented toward enhancing unconditional, positive emotional states of kindness and compassion), and “choiceless awareness” (open monitoring of mind wandering) compared to inexperienced controls (Brewer et al., 2011; Hofmann et al., 2011). Self-reported time on task during “mindfulness of breathing” was higher in experienced than in inexperienced participants, indicating less mind wandering with training (Holzel et al., 2007). In contrast, there was no correlation between the number of button presses indicating epochs of mind wandering during focused attention on the breath with years of practice or with high vs. low practice groups (Hasenkamp et al., 2012). In this study, participants recorded an average of one mind wandering per 80 s over a 20-min fMRI session, by pressing a button whenever they realized that their mind had wandered completely away from the breath.

### Default mode network activation

Many concepts of how meditation affects mind wandering derive from its association with the default mode network. A number of imaging studies have shown that a system of cortical areas increase their activation when the brain is not engaged in an externally defined task, and that the magnitude of increase correlates with the extent of mind wandering (Mason et al., 2007; Buckner et al., 2008). Although some variation occurs, the default network mostly includes medial brain structures, i.e., the ventral medial prefrontal cortex, the posterior cingulate/retrosplenial cortex, the inferior parietal lobe, the lateral temporal cortex, the dorsal medial prefrontal cortex, and the hippocampal formation (Buckner et al., 2008).

A majority of the studies on meditation and mind wandering have measured how fMRI activation and functional connectivity of the default mode network are related to mind wandering. Most of these describe trait differences in brain activation patterns arising from meditation, often showing decreased default mode network activation in experienced meditators compared to novices (Brewer et al., 2011; Sood and Jones, 2013).

Only a few studies have reported state changes, contrasting meditation with various control tasks in the same practitioners, but with varying results. Using rest as a control, Brefczynski-Lewis and coworkers showed activation of a large overlapping network of attention-related cortical regions during “concentration meditation” (focused attention with a simple visual stimulus), including frontal, parietal regions, lateral occipital cortex, and insula (Brefczynski-Lewis et al., 2007). Lazar and coworkers showed activation of dorsolateral prefrontal and parietal cortices, hippocampus/parahippocampus, temporal lobe, pregenual anterior cingulate cortex, striatum, and pre- and post-central gyri during mantra meditation coordinated with breath (Lazar et al., 2000). Generating a list of animals was used as control task. Engström and coworkers compared mantra meditation with silent repetition of a short semantic phrase as control and detected activation in bilateral hippocampus/parahippocampal formations, as well as bilateral middle cingulate cortex and bilateral precentral cortex (Engstrom et al., 2010). Interestingly, Manna and coworkers (Manna et al., 2010) described reduced activation of precuneus (a core default mode network area) compared to rest during meditation with focused attention on the breath, and increased activation during meditation with open monitoring of “any experiential or mental content” (Manna et al., 2010). None of the aforementioned studies assessed the extent of mind wandering.

### Aim and hypothesis

The aim of the present study was to determine whether nondirective meditation is conducive to default mode network activation. We hypothesized that accepting the spontaneous flow of thoughts, images, sensations, memories, and emotions as part of meditation, without any emphasis on reducing, monitoring, evaluating or directly relating to it, would increase mind wandering and activation of the default mode network, compared to practicing with more emphasis on control and a concentrative focus of attention. We therefore assessed whether practicing the same technique (Acem meditation) with different types of attentional focus would affect the subjective experience and the pattern of brain activation during meditation assessed by fMRI.

## Methods

### Ethics statement

The National Committee for Medical Research Ethics in Norway approved the study. Informed written consent was obtained from all participants before inclusion.

### Participants

Twenty-seven experienced practitioners of Acem meditation (18 men and 9 women) were recruited. All participants were regular practitioners (2 × 30 min daily) and had extensive experience with longer meditation periods, including participation in at least one 3-week long retreat. Twenty-four were right handed, ascertained by the Edinburg Handedness Inventory (Oldfield, 1971). Thirteen participants were excluded from final data analysis due to rigorous quality control; only participants with acceptable recordings from both fMRI sessions were included. Three were excluded because of reported sleep during the recording, two because of significant head motion (≥1 mm), one because of error in scanning protocol, and seven because of technical problems that lead to corruption of the fMRI images. Even though the head was securely fixed inside the headcoil according to standard procedure (using triangular shaped foam pads), minor involuntary movements were difficult to avoid during two 20-min recordings in a relaxed reclining condition. Thus, 14 practitioners (8 men and 6 women, 13 right handed), aged 28–61 years (mean 49, SD 9) with 9–38 years of meditation practice (mean 27, SD 9) were included in final data analysis. We included only experienced meditators in our study, since it takes extensive training to reliably distinguish between nondirective and concentrative practicing.

### fMRI meditation instructions

Details on nondirective meditation has been provided above (Box 3) and in previous publications (Ellingsen and Holen, 2008; Davanger et al., 2010). Participants were asked to perform Acem meditation in two separate runs of fMRI acquisition. In nondirective meditation the participants were instructed to repeat the meditation sound in a relaxed and effortless manner, in the same way as during home practice. Spontaneous mind wandering was neither prevented nor encouraged. In contrast, during concentrative practicing, the meditation sound was repeated in a more forceful manner, with strict regularity, in order to maintain the focus of attention on the sound, attempting to avoid mind wandering. As expected, mind wandering was not avoided completely, although more of the participants reported decreased mind wandering during concentrative practicing than in nondirective meditation. During data acquisition in the resting blocks (see below) participants were instructed to rest without repeating the meditation sound, allowing mind wandering where spontaneously occurring thoughts, images, sensations, memories, and emotions could emerge and pass freely.

### Experimental design

In order to establish a stable, relaxed resting control state, all participants meditated for 45–60 min before experimental recordings. Each practitioner was scanned in one session with one run of nondirective meditation and one of concentrative practicing (block design), presented in randomized order. In each run the practitioners performed a sequence of four meditation blocks lasting 3, 5, 4, and 3 min respectively, interspersed with five resting blocks lasting 1 min each. Block length was varied in order to avoid “false” fMRI activation induced by expectation. All subjects were scanned with eyes closed. Concentrative practicing and rest were used as contrasts for nondirective meditation. This would minimize the possible effect of underlying traits in the subjects, each subject serving as his or her own control. Immediately following each scanning run, all participants were asked to complete a questionnaire assessing their meditation experiences: extent of mind wandering compared to regular home practice, whether they became drowsy or briefly fell asleep, and to what extent the sound from the MRI scanner was disturbing. They also confirmed whether they had been able to carry out the meditation tasks.

### Data acquisition

Structural and functional scanning was performed using a 3T Philips Intera scanner (Philips Medical, Best, The Netherlands) with an 8-channel SENSitivity Encoding (SENSE) head-coil (InVivo, Gainsville, FL, USA). Using BOLD-sensitive imaging, a total of 400 volumes was acquired for each run with a gradient-echo echo-planar-imaging pulse sequence. Each volume consisted of 44 contiguous axial slices, with the following scan parameters: SENSE-reduction factor = 2.2, *TR* = 3000 ms; flip angle = 90°; *TE* = 35 ms; *FOV* = 230 mm; slice thickness = 2.5 mm; matrix = 64 × 64 giving an in-plane resolution of 3.6 × 3.6 mm^2^. Also a high-resolution T1-weighted image series was collected using a three-dimensional magnetization-prepared rapid gradient echo sequence (MP-RAGE) consisting of 182 contiguous sagittal slices of 1.2-mm thickness with an in-plane resolution of 1 × 1 mm. For analysis, all images were reconstructed to 1 mm^3^.

### Data analysis

Imaging data were analyzed using FSL 4.0 (Analysis Group, FMRIB, Oxford, UK; www.fmrib.ox.ac.uk/fsl/). First, non-brain tissue was removed from the T1-weighted anatomical image-series using the Brain Extraction Tool (Smith, 2002). The resulting images were transformed non-linearly to the MNI152 1 × 1 × 1 mm template (Montreal Neurological Institute, Montreal, QC, Canada), and motion corrected with the median volume of each run as reference using the FNIRT algorithm (Andersson et al., 2007). Then each functional run was co-registered to the corresponding anatomical T1-weighted image-series and transformed into MNI152 space by the transformation matrix obtained from the T1-weighted images. The functional data was smoothed by a 6 mm full-width at half-maximum (FWHM) Gaussian filter, and a temporal high-pass filter with a cut-off time of 350 s.

The two-level random effects statistical analysis of the fMRI data was carried out using Bayesian estimation techniques with FEAT (Smith et al., 2004). Conditions were modeled according to a boxcar stimulus function convolved with a two-gamma hemodynamic response function (Boynton et al., 1996). The first minute of each meditation block was excluded from the analysis by modeling it as non-effect, as meditation activations take time to build up (Davanger et al., 2010). The effect of each condition was estimated according to a general linear model (Friston et al., 1995). A whole-brain analysis was performed using mixed effects FLAME-1 algorithms (Beckmann et al., 2003). Statistical thresholds for contrasts nondirective meditation > rest, and concentrative practicing > rest were set to *p* < 0.05, family wise error rate was corrected using cluster-level interference by setting cluster forming threshold at *z* > 3.0 (*p* < 0.0027). For the contrast nondirective meditation > concentrative practicing it was set to *p* < 0.05 and cluster forming *z* > 2.3 (*p* < 0.0214). To increase sensitivity, the threshold was set less stringently for the latter comparison, because the expected difference between two similar conditions is usually smaller and the variability greater than for respective comparisons with rest. For all three contrasts, correlation analysis with years of experience as independent variable was performed in FEAT using FLAME-1 algorithm. Years of experience was defined as an extra environmental variable for all three contrasts. Brain areas were identified by FSL atlases and other relevant sources for functional data as referenced.

### Statistical analysis of questionnaire data

A post-scan behavior questionnaire comprised three questions (translated from Norwegian): (1) How disturbing was the scanner sound in the background: 0 = not at all, 1 = some, 2 = much. (2) What was the extent of mind wandering compared to regular meditation outside the scanner: 0 = less, 1 = similar, 2 = more. (3) Did you become drowsy or fall asleep: 0 = wakeful, 1 = drowsy, 2 = fell asleep. The questionnaire data were analyzed in Microsoft Excel (Microsoft Corporation, Redmond, WA, USA). Fisher's exact test was performed to assess whether mind wandering, drowsiness and disturbance by scanner depended on the mode of practicing (nondirective vs. concentrative) in 2 × 2 tables, excluding table lines with zero-cells. As described below, participants who fell asleep during scanning, were excluded from further analyses.

## Results

### Behavioral data

Data from a brief questionnaire administered immediately after each fMRI recording indicated a trend for less mind wandering with concentrative practicing compared to regular meditation. Even though the meditation blocks were short and the number of participants small, a larger number experienced less mind wandering during concentrative practicing than during nondirective meditation, whereas the numbers of participants who were wakeful/drowsy and disturbed some/much by noise were similar during nondirective and concentrative practicing, respectively (Table 1). A majority spontaneously remarked that concentrative practicing was effortful and tiring, although it was not an item in the questionnaire.

**Table 1:** **Meditation experience during scanning assessed by post-scan questionnaire**.

	**Nondirective meditation**	**Concentrative practicing**	***P*-value**
**MIND WANDERING**			
Less	8	12	
Similar	6	2	0.09
More	0	0	
**WAKEFULNESS**			
Wakeful	10	9	
Drowsy	4	5	0.29
**DISTURBED BY NOISE**			
Not at all	1	0	
Some	8	10	
Much	5	4	0.23

*For mind wandering, numbers denote participants experiencing less or similar mind wandering in the scanner compared to regular meditations. P-values were assessed by Fisher's exact test, as described in Methods*.

### fMRI data

The fMRI assessments showed that nondirective meditation activated several regions of the cerebral cortex as well as subcortical structures significantly more than during resting. However, compared to nondirective meditation, during concentrative practicing fewer areas were activated more than at rest. Some regions in the right temporal lobe were activated significantly stronger during nondirective meditation than concentrative practicing. The activated areas for each contrast are detailed below. There was no correlation between activation and years of meditation experience.

### Nondirective meditation

**Figure 1:**
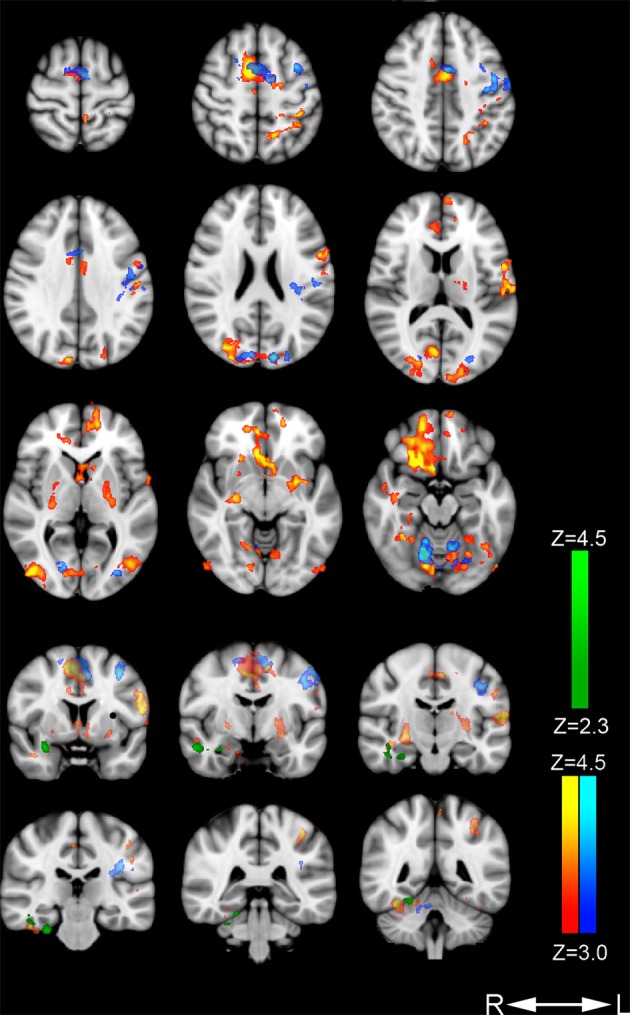
**Areas with increased cerebral activation**. Color-coded regions show activation above threshold in the following contrasts: *Nondirective meditation > rest* (red-yellow), *concentrative practicing > rest* (dark blue-light blue), and *nondirective meditation > concentrative practicing* (dark green-light green). Activations are superimposed on MNI template (Montreal Neurological Institute).

Increased signal for the contrast *nondirective meditation > rest* was found in several regions, including orbitofrontal, motor, somatosensory, visual, association, and limbic areas (Figure 1; Table 2). Notably, nondirective meditation increased activity in the *prefrontal* cortex, showing a large cluster with the point of maximal activation in the straight gyrus, covering a large part of the right orbitofrontal cortex as well as medial prefrontal areas. Also the *anterior cingulate cortex*, parts of the parietal lobe (*posterior cingulate cortex, precuneus, anterior/inferior parts of the lateral parietal lobe*) and the temporal lobe (*inferior and medial temporal lobe, hippocampus, amygdala*) were activated more than at rest.

**Table 2:** **Cerebral regions with increased activity: nondirective meditation > rest**.

**Nondirective meditation > Rest**								
**z-threshold: 3.0 and Cluster forming P-threshold: 0.05**								
**Anatomical location**	**Hemisphere**	**Lobus**	***X***	***Y***	***Z***	**z-score**	**Cluster size**	***BA***
Straight gyrus (frontal lobe)	R	Frontal	22	36	−21	4.86	25,554	11
Middle occipital gyrus (secondary visual cortex)	R	Occipital	24	−86	27	4.66	18,659	18
Inferior occipital gyrus (secondary visual cortex)	R	Occipital	36	−83	0	4.54	2754	19
Middle occipital gyrus (secondary visual cortex)	R	Occipital	30	−86	20	4.5	18,659	18
Premotor cortex (lateral), (supplementary motor area)	R	Frontal	7	−74	−14	4.35	18,659	17
Precentral gyrus (premotor area, Broca's area)	L	Frontal	−56	5	17	4.32	8340	6, 44
Superior parietal lobule (secondary sensorimotor cortex)	L	Parietal	−14	−56	55	4.24	8340	5, 7
Postcentral gyrus, superior temporal gyrus	L	Parietal	−61	−15	14	4.17	8340	1, 2, 3
Superior parietal lobule (secondary sensorimotor cortex)	L	Parietal	−19	−60	47	4.12	8340	5, 7
Inferior temporal, fusiform, parahippocampal gyrus	R	Temporal	44	−26	−27	4	3196	20
Hippocampus	R	Temporal	27	−17	−6	3.96	3196	N/A
Insula (posterior)	L	Insula	−37	−2	−7	3.91	3173	14
Middle occipital gyrus (secondary visual cortex)	L	Occipital	−20	−74	−12	3.85	18,659	18
Inferior temporal and fusiform gyrus	R	Temporal	38	−27	−32	3.59	3196	20
Anterior cingulate cortex	R	Frontal	8	37	10	3.58	25,554	32
Posterior cingulate cortex	R	Frontal	8	6	31	3.54	25,554	24
Anterior cingulate cortex	L	Frontal	−3	42	−3	3.54	25,554	32
**SUBCORTICAL**								
Putamen	L	Sub-cortical	−27	−2	−8	4.22	3173	
Nucleus accumbens	L	Sub-cortical	−7	10	−9	4.16	25554	
Pallidum	L	Sub-cortical	−24	−11	2	3.69	3173	
Nucleus caudatus	L	Sub-cortical	−11	15	3	3.6	25554	
Amygdala	R	Sub-cortical	23	−5	−21	3.47	3196	
Thalamus	L	Sub-cortical	−16	−15	15	3.43	3173	
Pallidum	R	Sub-cortical	22	−5	−2	3.39	3196	
Putamen	R	Sub-cortical	25	4	−5	3.15	3196	

*The analysis was carried out using whole brain analysis with z-threshold = 3.0 and cluster forming p-threshold: 0.05. Coordinates in MNI coordinates (Montreal Neurological Institute) (R, right; L, left; BA, Brodmann's area)*.

Large clusters were also detected in the occipital lobe covering vision areas in the middle occipital gyrus and striate cortex. In the posterior part of the frontal lobe, activation occurred in *primary* and *supplementary motor* areas of the left hemisphere, extending into Broca's area.

In the left parietal lobe, *sensorimotor* and *secondary sensory* regions including part of the precuneus were activated. There was no change in Wernicke's receptive speech area.

In the right *temporal* lobe, three clusters were found: the fusiform cortex/inferior temporal gyrus/parahippocampal gyrus including the visual processing and facial areas, the hippocampus, and the amygdala.

In the *cingulate* cortex, separate clusters in the right and left anterior regions were activated, as well as in the right posterior regions. Activated clusters were also seen in two non-cortical regions: In the left *basal ganglia* (putamen, globus pallidus, and the nucleus accumbens), and in a right and a left *cerebellar* region.

The opposite contrast, *nondirective meditation < rest*, showed no positive activation.

### Concentrative practicing

The contrast *concentrative practicing > rest* revealed significant activation in three regions (Figure 1; Table 3). *Motor area* activation was present in the posterior part of the middle frontal gyrus/premotor cortex, precentral gyrus, the primary motor cortex, and the supplementary motor area/pre-motor cortex. In *visual areas*, we observed activation of the middle and inferior occipital gyrus/lateral occipital cortex, the occipital fusiform gyrus, and the intracalcarine/visual and the occipital pole/visual cortices. Lastly, one cluster was activated in the dorsal aspect of the anterior *cingulate* cortex, bilaterally. No parietal or temporal clusters were seen during concentrative practicing.

**Table 3:** **Cerebral regions with increased activity: concentrative practicing > rest**.

**Concentrative practicing > Rest**								
**z-threshold: 3.0 and Cluster forming P-threshold: 0.05**								
**Anatomical location**	**Hemisphere**	**Lobus**	***X***	***Y***	***Z***	**z-score**	**Cluster size**	***BA***
Middle occipital gyrus (secondary visual cortex)	L	Occipital	−13	−89	24	4.17	2086	18
Middle frontal gyrus, premotor cortex	L	Frontal	−36	5	49	4.1	6185	6
Supplementary motor cortex, premotor cortex	L	Frontal	−6	−3	61	4.02	6151	6
Precentral gyrus, primary motor cortex	L	Frontal	−39	−14	41	3.9	6185	4
Middle frontal gyrus, primary motor cortex	L	Frontal	−34	7	55	3.88	6185	6
Middle frontal gyrus, premotor cortex	L	Frontal	−48	−20	37	3.87	6185	3
Calcarine cortex, primary visual cortex	R	Occipital	17	−74	5	3.85	1874	17
Middle frontal gyrus, premotor cortex	L	Frontal	−37	6	58	3.83	6185	6
Calcarine cortex, primary visual cortex	R	Occipital	15	−87	19	3.73	1874	17
Anterior cingulate cortex, dorsal part	R	Frontal	4	11	31	3.63	6185	24
Anterior cingulate cortex, dorsal part	L	Frontal	−1	13	34	3.49	6151	24
Inferior occipital gyrus, secondary visual cortex	L	Occipital	−21	−75	−11	3.46	2049	18
Supplementary motor cortex, premotor cortex	R	Frontal	7	6	53	3.42	6151	6

*The analysis was carried out using whole brain analysis with z-threshold = 3.0 and cluster forming p-threshold: 0.05. Coordinates in MNI coordinates (Montreal Neurological Institute) (R, right; L, left; BA, Brodmann's area)*.

The opposite contrast, *concentrative practicing < rest*, showed no positive activation.

### Nondirective meditation vs. concentrative practicing

The contrast *nondirective meditation > concentrative practicing* (Figure 1; Table 4) revealed higher activation of several areas in the temporal lobe: middle and inferior temporal gyrus, fusiform gyrus, amygdala, and parahippocampal gyrus.

**Table 4:** **Cerebral regions with increased activity: nondirective meditation > concentrative practicing > rest**.

**Nondirective meditation > Concentrative practicing**								
**z-threshold: 2.3 and Cluster forming P-threshold: 0.05**								
**Anatomical location**	**Hemisphere**	**Lobus**	***X***	***Y***	***Z***	**z-score**	**Cluster size**	***BA***
Middle temporal gyrus	R	Temporal	52	−6	−22	3.25	3616	21
Parahippocampal gyrus	R	Temporal	28	−24	−32	3.22	3616	
Inferior temporal gyrus, fusiform gyrus	R	Temporal	36	−2	−20	3.13	3616	20
Inferior temporal gyrus, fusiform gyrus	R	Temporal	47	−29	−19	3.12	3616	20
**SUBCORTICAL**								
Amygdala	R	Subcortical	31	2	−19	3.45	3616	

*The analysis was carried out using whole brain analysis with z-threshold = 2.3 and cluster forming p-threshold: 0.05. Coordinates in MNI coordinates (Montreal Neurological Institute) (R, right; L, left; BA, Brodmann's area)*.

The opposite contrast, *nondirective meditation < concentrative practicing*, showed no positive activation.

## Discussion

The present study sought to investigate state effects of nondirective meditation either compared to rest or to concentrative practicing in participants with long-term experience of Acem meditation. Results are consistent with the notion that nondirective meditation involves more extensive activation of the default mode network, including brain areas associated with episodic memories and emotional processing.

### Default mode network activation

Compared to rest, nondirective meditation increased activation within all cortical areas defining the default mode network (Buckner et al., 2008), including the ventral medial prefrontal cortex, the posterior cingulate/retrosplenial cortex, the inferior parietal lobe, the lateral temporal cortex, the dorsal medial prefrontal cortex, and the hippocampal formation (Figure 1, Table 2). The pattern of activations was similar to that associated with mind wandering in a recent study of meditation with focused attention on breath, including posterior cingulate cortex, medial prefrontal cortex, posterior parietal and temporal cortex, and the hippocampus (Hasenkamp et al., 2012). In contrast, the control task of concentrative practicing in the present study seemed to have little effect on default mode network activation, including only the anterior cingulate cortex when compared to rest (Figure 1, Table 3). However, direct comparison of nondirective meditation with concentrative practicing gave only temporal clusters, including parahippocampal areas and amygdala. These observations indicate that the extent of default mode network activation during concentrative practicing probably lies somewhere between nondirective meditation and rest: slightly more than in rest, but evidently not enough to yield significant clusters in most default mode areas. This interpretation is consistent with the trend of less mind wandering reported in concentrative practicing compared to nondirective meditation (Table 1).

Our results corroborate previous findings that suggest increased default mode network activation during meditation. Experienced Vipassana meditators (focused attention on breath) showed stronger activation of the anterior cingulate cortex and the dorsal medial prefrontal cortex than control subjects (Holzel et al., 2007). During resting state, practitioners of “brain-wave vibration meditation” (meditative movement) had greater functional connectivity within the default mode network in the medial prefrontal cortex than controls (Jang et al., 2011). Performing Transcendental Meditation (another form of nondirective meditation) gave higher alpha1 EEG activity in midline cortical regions that overlapped with the default mode network (Travis et al., 2010).

Our findings regarding default mode network activation are in contrast with the prevailing view of practices with reference to mindfulness or Buddhist traditions, as recently reviewed (Sood and Jones, 2013). For example, experienced practitioners of “concentration” (focused attention), “loving-kindness” (exercise oriented toward enhancing unconditional, positive emotional states of kindness and compassion), and “choiceless awareness” (open monitoring of mind wandering) showed decreased default mode network activation compared to inexperienced controls (Brewer et al., 2011; Hofmann et al., 2011), and experienced Zen meditators had weaker connectivity between the medial prefrontal cortex and several other default mode network nodes (Taylor et al., 2013). These practices are described as “a training of attention away from self-reference and mind-wandering, and potentially away from default-mode processing” (Brewer et al., 2011). Reduced activation of a core default mode network component (precuneus) has been described in experienced Buddhist monks during focused attention on the breath, whereas the same area had larger activation than rest during open monitoring of “any experiential or mental content” (Manna et al., 2010).

Altogether, present and previous results suggest that the relationship with type of practice and years of experience is more complex than the presumption that “meditation reduces mind wandering and default mode network activation.” Our observations indicate a differential effect related to the relaxed focus of attention in nondirective meditation vs. concentrative practicing, actively trying to avoid mind wandering.

### Prefrontal and temporal functions: attention and emotional processing

Across several forms of meditation, regulation of attention has consistently been linked to increased activity within the anterior cingulate cortex and the prefrontal cortex (Lazar et al., 2000; Kubota et al., 2001; Cahn and Polich, 2006; Holzel et al., 2008; Chiesa and Serretti, 2010; Davanger et al., 2010; Engstrom and Soderfeldt, 2010; Manna et al., 2010; Hasenkamp et al., 2012). Some studies have indicated that in meditation, the dorsal anterior cingulate cortex is most probably involved in attention and in discriminating between relevant and distracting thoughts, whereas the ventral aspect may serve as a link between emotional processing and autonomic regulation in the hypothalamus (Ongur et al., 1998; Johansen-Berg et al., 2008).

In the present study, the prefrontal cortex was activated in a large orbitofrontal and medial cortex cluster (included in the straight gyrus cluster, frontal lobe) and in an anterior cingulate cluster during nondirective meditation (Figure 1, Table 2). In contrast, orbitofrontal and medial areas of the prefrontal cortex (excluding anterior cingulate cortex) were not activated during the control task of concentrative practicing (Figure 1, Table 3). As suggested by observations from other contexts (Etkin et al., 2011), we speculate that part of the activation in these areas might be associated with emotional processing related to mind wandering, which would be an interesting topic for future research. A significant difference between nondirective meditation vs. the control conditions of either rest or concentrative practicing was activation of the anterior hippocampus and amygdala (Figure 1, Tables 2–4). In addition to spatial orientation, these areas have been associated with memory and emotional processing (Fanselow and Dong, 2010).

Hippocampus activation has been associated with mind wandering by detailed temporal analysis of meditation with focused attention on breath (Hasenkamp et al., 2012); as noted above, it is a core component of the default mode network (Buckner et al., 2008). Concomitant activation of hippocampus and amygdala has been reported in two previous studies of silent nondirective mantra meditation and relaxation response (Lazar et al., 2000; Engstrom et al., 2010). In contrast, amygdala activation was reduced in a study of mindfulness meditation (a breath-focused attention task) (Goldin and Gross, 2010), and in loving-kindness meditation (Brewer et al., 2011). Whereas isolated amygdala activation may indicate psychological strain in post-traumatic stress disorder (Hughes and Shin, 2011), concomitant activation with the dorsolateral prefrontal cortex, anterior cingulate cortex, and the hippocampus may possibly serve to modify stressful emotional memories (Phillips et al., 2003; Shin et al., 2006). On the other hand, activation of amygdala has been correlated with subjective effort (Dyck et al., 2011). Further investigations are needed to determine the function of concomitant activation of hippocampus and amygdala in meditation.

## Limitations

Some of the present experimental conditions differ significantly from actual meditation and may limit generalizability of the results. A major issue was that the participants meditated lying supine in the scanner (as opposed to sitting). As emphasized in a recent source of mindfulness-based cognitive therapy (Segal et al., 2013), reclining with eyes closed predisposes for relaxation, drowsiness, and even brief episodes of sleep, e.g., during body scan (page 156). A consequence of this was a tendency of subtle, involuntary movement during the two 20-min fMRI recordings, despite fixing the head according to standard procedure. Thirteen out of 54 original scans (24%) were excluded, a similar rate as observed in a previous study of mantra meditation (Engstrom et al., 2010). Since data from nondirective meditation and concentrative practicing was analyzed by pair-wise comparison, the whole data set of a participant was removed if one of the recordings was excluded. Thus, exclusion rate seems twice as high as actual problems with recordings. Nevertheless, the number of exclusions was unusually high, and may limit the generalizability of the findings. The low number included in final analyses is a limitation *per se*.

A factor that may have influenced activation patterns during meditation was noise from the scanner, which might explain less mind wandering than “in usual meditation” in more than 50% of the participants (Table 1). However, there was a strong trend for less mind wandering during concentrative practicing than during nondirective meditation, indicating their effort to maintain attention with the meditation sound. This suggests that the meditation tasks were largely performed according to instructions. It is also possible that the participants could have been biased toward rating mind wandering more frequently during nondirective meditation, as this was their regular practice. In summary, data from the questionnaire suggest that results from the included participants may be relevant for understanding mechanisms related to mind wandering, although external study conditions varied significantly from actual meditation outside the scanner.

## Conclusion

The present study demonstrates that nondirective meditation induces more extensive default mode network activation than rest. Even though a core characteristic of the practice is a relaxed focus of attention that accepts mind wandering as part of the process, it is a paradox that the active task of effortless mental repetition of a meditation sound yields larger default mode network activation than the passive task of simply resting. This observation suggests that the nondirective meditation task involves a minimal level of cognitive effort, which is often emphasized as an important characteristic of successful practicing across different types of techniques used for health and wellness, including focused attention, open monitoring, and nondirective meditation. The study also shows that the control task of concentrative practicing of the same technique (Acem meditation), performed with an effort to reduce mind wandering, reduced the extent of default mode network activation compared to nondirective meditation, but not below the level of resting.

Altogether, our findings support the notion that nondirective meditation is conducive for default mode network activation. They also indicate that this activation is related to the relaxed focus of attention, which allows spontaneous thoughts, images, sensations, memories, and emotions to emerge and pass freely, accepting them as part of the meditation process. Since the relaxed focus of attention is a core component of several practices, we speculate that mental activities associated with default mode network activation, may be essential for state and trait effects. Further research is needed to determine whether this activation is associated with retrieval of episodic memories and emotional processing during nondirective meditation.

### Conflict of interest statement

Svend Davanger, Øyvind Ellingsen, and Are Holen perform voluntary work for Acem School of Meditation, an international not for profit organization. The other authors declare that the research was conducted in the absence of any commercial or financial relationships that could be construed as a potential conflict of interest.
